# Business Notes

**Published:** 1870-01

**Authors:** 


### BUSINESS NOTES.

We issue this number of the *Bistoury*, as is our custom, in advance of the holidays, in order to give our advertisers an opportunity of informing the public where to look for holiday gifts.

FOR book binding and marking of gift books in gilt, go to Kies in *Advertiser* building.

FOR the fancy articles usually kept in a first-class drug store, go to KELLOGG & CONVERSE, 145 Water Street.

CARPETS and house furnishing goods can be found at DURLAND & PRATTS, 132 Water Street.

SHOULD you desire to treat yourself to a handsome new suit go to BOWER & ROMER, at 149 Water Street.

THOMAS D. BALDWIN, at 115½ Water Street, displays a handsome line of gentlemen's cloths. He has an excellent cutter and is doing nice work.

AT E. H. COOK & Co's you will find skates of all sizes and patterns, suitable for girls or boys. Surprise and gratify your little ones by making your selection from this stock.

S. T. STEELEY advertises $30.000 worth of dress goods at, and below cost, at his store, 36 Opera House Block. This is a splendid opportunity to buy goods cheap.

MANY handsome presents can be selected in the way of household goods, such as easy chairs, fancy parlor chairs, canopy bedsteads, &c. For these go to HURBELL, the furniture man, in Union Block, Water Street.

BARNEY, STOWELL, & DAVIS have just opened an endless variety of fancy goods for ladies and children, expressly selected for the holidays, at their beautiful store in Opera House block. If you don't know what sort of a gift to select they will assist you in deciding.

COLLINGWOOD & STRANG have on exhibition a larger stock of holiday goods than usual. Henry has just returned from New York with everything new in the line of jewelry, solid silver and plated wares, bronzes, watches, charms, and an endless variety of fancy articles, useful and ornamental. Go make your selections early before the handsomest of the articles are taken away.

FOR eatables—the necessaries of the Christmas feast, go to URI MILLS & SON, on Lake Street, and see one of the largest and handsomest grocery establishment in the country. You will here find everything in the market, for table use.

J. J. THRO, the "Post Office Grocer" is constantly receiving foreign fruit, canned fruit, &c., &c., for the holiday season. Call on him at his store next the Post Office.

THE HALL BROTHERS, at 128 Water Street, offer an unusually large stock of gift books and fancy articles. Call and see the great variety of cromos :—They will make suitable and handsome gifts for either ladies or gentlemen. You will also find a large collection of stereoscopic views, chess men, domino-boards, &c., &c. We have not the space to enumerate half the articles displayed in this house for the holidays —go and see for yourself.

